# Damage due to ice crystallization

**DOI:** 10.1038/s41598-025-86117-5

**Published:** 2025-01-16

**Authors:** Menno Demmenie, Paul Kolpakov, Boaz van Casteren, Dirk Bakker, Daniel Bonn, Noushine Shahidzadeh

**Affiliations:** 1 Van der Waals-Zeeman institute, Institute of Physics, University of Amsterdam, Science Park 904, 1098 XH Amsterdam, The Netherlands; 2 Van ’t Hoff Institute for Molecular Sciences, University of Amsterdam, Science Park 904, 1098 XH Amsterdam, The Netherlands

**Keywords:** Fluids, Fluid dynamics, Applied physics

## Abstract

The freezing of water is one of the major causes of mechanical damage in materials during wintertime; surprisingly this happens even in situations where water only partially saturates the material so that the ice has room to grow. Here we perform freezing experiments in cylindrical glass vials of various sizes and wettability properties, using a dye that exclusively colors the liquid phase; this allows precise observation of the freezing front. The visualization reveals that damage occurs in partially water-saturated media when a closed liquid inclusion forms within the ice due to the freezing of the air/water meniscus. When this water inclusion subsequently freezes, the volume expansion leads to very high pressures leading to the fracture of both the surrounding ice and the glass vial. The pressure can be understood quantitatively based on thermodynamics which correctly predicts that the crystallization pressure on the inclusion boundary is independent of the volume of the liquid pocket. Finally, our results also reveal that by changing the wetting properties of the confining walls, the formation of the liquid pockets that cause the mechanical damage can be avoided.

## Introduction

Most of us have faced at least once in our life the catastrophic situation of having forgotten a bottle of liquid in the freezer, and found it broken the next day. The freezing of water in confined spaces is an important issue for various industries, including infrastructure maintenance^1,2^, cryopreservation^3–5^, agriculture^6,7^, and art conservation^8,9^; in all these cases, frost is known to cause frequent and costly damage. The most common explanation for that is based on the unusual property of water in liquid and solid states in comparison to other materials: In the liquid state, water molecules are more densely packed compared to the their arrangement in the crystalline lattice of ice. Consequently, water undergoes volumetric expansion when transformed into ice, potentially causing high pressures on confining walls and inducing mechanical damage^10–15^. However, this explanation could fall short as, for instance, mechanical damage is also reported in situations where water does not completely saturate the confined space and still has room to expand. This is especially true in situations where the ice is confined in one direction but can expand in another direction; think for instance of a glass bottle in the freezer being only half-filled with liquid. One would then expect the ice to be able to grow where there is room for it to expand without inducing mechanical damage. Nonetheless, mechanical damage is indeed observed in these cases. While recent studies have mainly concentrated on frost damage in microstructures and porous materials, such as frost heave and ice lens formation caused by cryosuction^16–18^, this work addresses the underlying damage mechanisms occurring at the macroscale.

**Figure 1:**
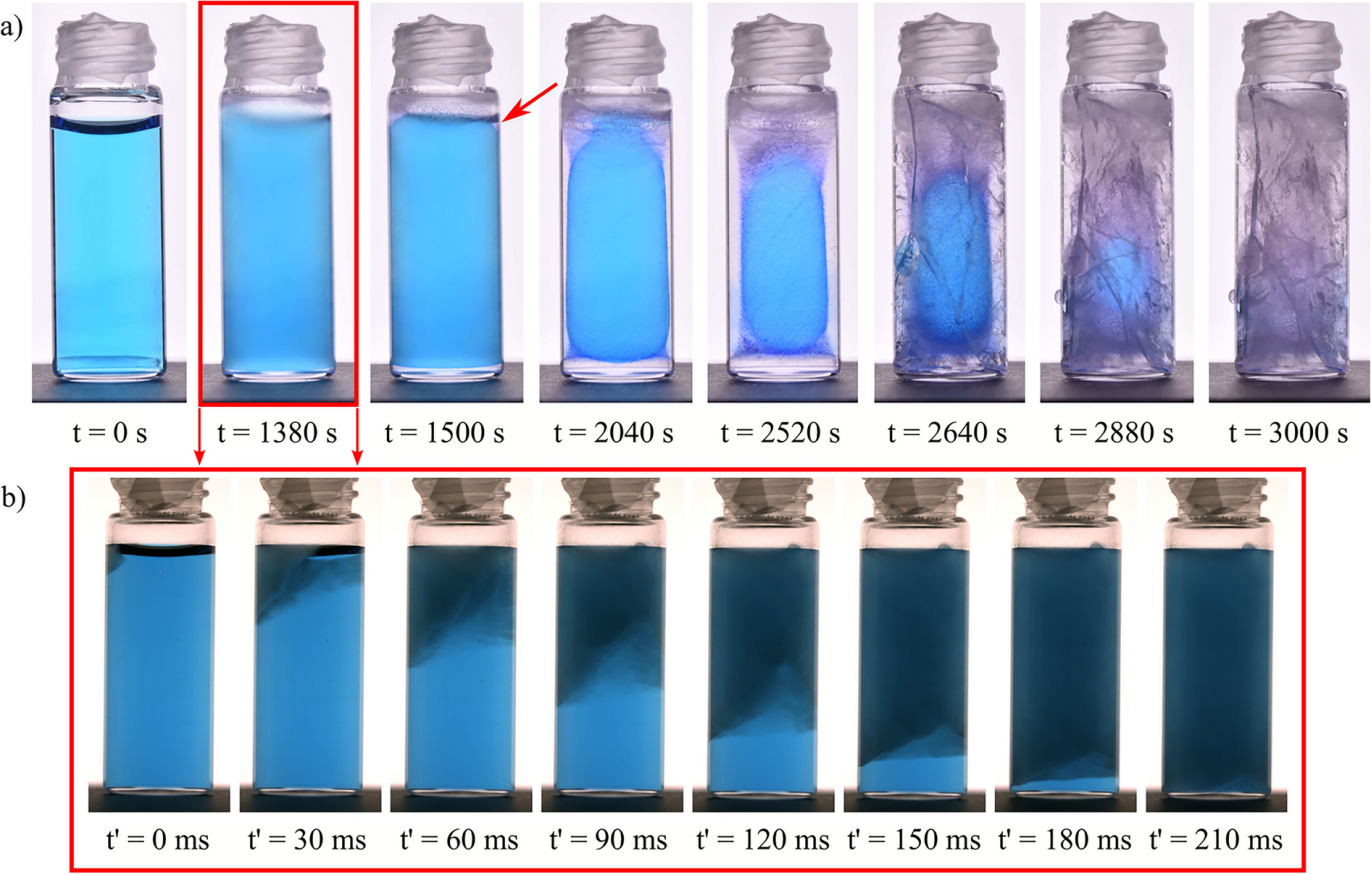
Snapshots of key moments in the freezing process of 4 mL of confined water at an ambient temperature of $-30$ $^\circ$C. Red arrow indicates the location where bulk crystallization initiates, at the contact line of the concave meniscus **(a)** Example of two-step crystallization after sufficient supercooling of the liquid. **(b)** Detailed chronology of the dendritic crystallization occurring after 1380 seconds in panel a, recorded at a frame rate of 2000 fps. From this sequence of images we obtain a dendritic crystallization velocity of $\sim 15$ cm/s, comparable to literature values^19,20^.

In this paper, we investigate ice crystallization in ’open’ confinements in cylindrical glass vials of different sizes and wetting properties, partially saturated with water. Our results reveal the major role of the formation of a liquid pocket, or liquid inclusion, within the ice and its subsequent transformation to ice in causing the observed mechanical damage. Our results show that ice nucleation and growth typically initiate at the free surface of a concave air/water meniscus if a hydrophilic glass vial is used. As the ice grows, the meniscus freezes before the bulk. Because of the expansion upon crystallization, the remaining liquid water can escape toward the air in the early stage, as the meniscus is not completely frozen. This phenomenon has previously been described as the origin of growing ice spikes^21^. When the outflow of water causes the opening to remain liquid, a crystalline hollow spike can grow upwards due to the partial wetting behavior of water on ice^22,23^. The optimal temperature for growing ice spikes appears to be around $-7$ $^\circ$C. Our experiments, conducted at $-30$ $^\circ$C, made the growth of ice spikes highly unlikely^24^. Hence, we observed the emergence of one ice spike in more than 120 experiments.

When the meniscus is completely frozen, it induces the entrapment of a liquid water pocket in the heart of the growing ice. The further transformation of this entrapped liquid volume into ice in the confined space leads to the fracture of both solid materials, i.e., the ice and the glass container. The latter can be understood mechanically and thermodynamically on the basis of an old paper by Gulida and Lifshitz^25^ who consider the opposite case of a liquid that melts locally within a solid. For a usual solid-liquid transition, the liquid occupies more space than the solid, and so a stress in the solid results from the transformation.

A hydrophobic treatment of the glass containers suppresses the curved shape of the meniscus to a flat one, shifting the ice nucleation point to the bottom of the glass. As a result, the probability of liquid pocket formation during the total phase transition is drastically reduced. Such treatment could be used as a potential mitigation strategy for freezing-induced damage in confined spaces. Our study also reveals that the degree of supercooling of the liquid before ice nucleation has a significant effect on the kinetics of ice crystallization and the potential for damage. When cooling from the outside, ice crystallization can occur in two stages: first, the formation of fast-growing dendritic ice on the glass wall, followed by the formation of a bulk ice crystal. Interestingly, this two-step process results in a significant number of entrapped air bubbles within the ice, which seem to act as pressure reservoirs and thereby reduce the probability of the container breaking.

**Figure 2:**
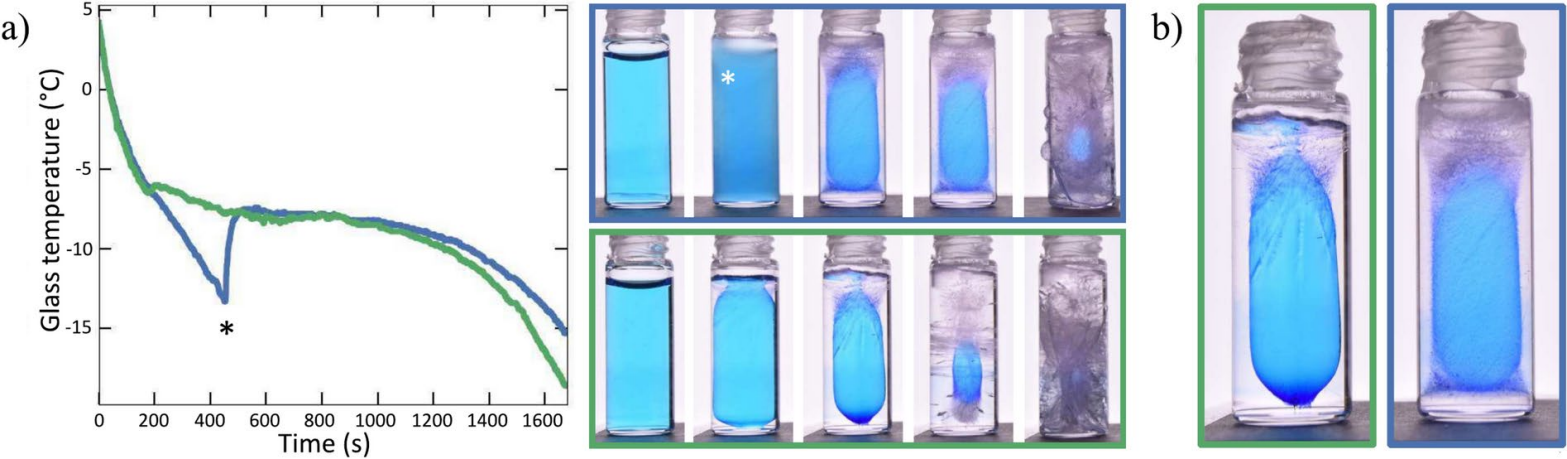
**(a)** Measured glass temperature of 2 containers. In blue, a sample that exhibited supercooling ($\Delta T \approx 6^\circ C$) whereas the green sample did not. The occurrence of supercooling goes hand in hand with dendritic crystallization, as indicated by an asterisk. **(b)** Magnified view of the middle image of the panel in Fig. (a), at the moment of complete meniscus freezing. The difference in the amount of entrapped air bubbles is clearly visible at the bottom of the liquid inclusions. The sample in the green frame shows transparent bulk ice, whereas the sample in the blue frame, which exhibited dendritic crystallization contains numerous entrapped air bubbles.

## Results

**Figure 3:**
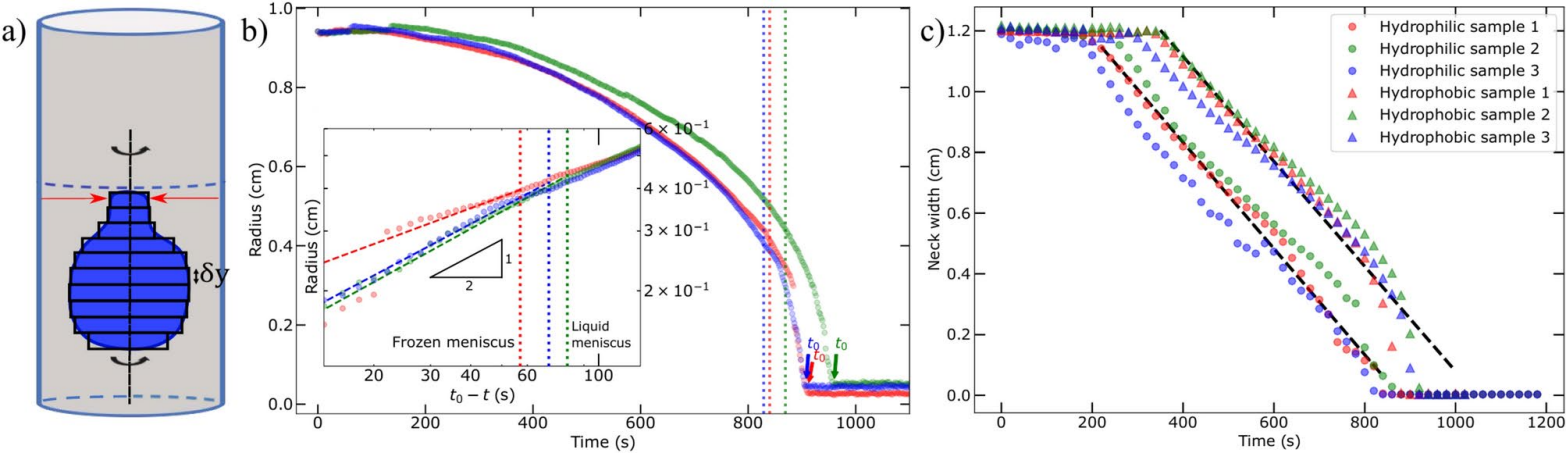
**(a)** Body of revolution method to determine the volume of the liquid inclusion. $\delta y$ has the thickness of one pixel and is therefore limited by the camera resolution. Red arrows show the position of the measured neck width, close to the meniscus. **(b)** Equivalent spherical radius decrease of a liquid inclusion in three experiments in a vial with an inner radius of 7.35 mm. The red symbols are from an experiment in which the glass container fractured at 885 seconds. Dotted vertical lines depict the time at which the meniscus freezes, closing off a route for water to escape. The discontinuity in the red data at $\sim$ t = 885 sec, is the moment of glass fracture. The unbroken vials show a continuous radius decrease in time (blue and green curves). The inset zooms in on this moment on double logarithmic scale. Herein, the x-axis is inverted (translated to $t_0 - t$, with $t_0$ the moment no more liquid is observed) to investigate the propagation rate of the ice front. Vials that did not fracture follow the expected relation ($r \sim \sqrt{t_0-t}$) for diffusion limited freezing of a liquid pocket. The red data, representing a vial that fractured, deviates from this relation and shows a clear discontinuity at the moment of fracture. **(c)** Decrease in neck width of three hydrophilically (dots) and hydrophobically (triangles) treated recipients. In hydrophobic bottles, the meniscus freezes at a later stage, although the velocity ($\sim 0.017$ mm/s) remains similar, as indicated by the fit of the slopes with the black dashed lines.

Fig. 1a shows a series snapshots of important moments in the freezing process at $-30$ $^\circ$C. The water was initially at room temperature. After 1380 seconds of cooling at $-30$ $^\circ$C, the sample first turns turbid due to a rapid dendritic growth of ice. Fig. 1b presents a fast sequence of snapshots taken by the rapid camera of the initial stages of the dendritic ice crystallization. In all experiments that exhibit dendritic crystallization, ice dendrites initiate at liquid meniscus. In recent decades, the influence of triple line (water/air/glass contact line) nucleation has been subject of controversy. Droplets freezing on extremely smooth, clean and homogeneously cooled silicon wafer substrates do not favor nucleation at the triple line^26^. Conversely, in environments with higher surface roughness, nucleation generally starts at the triple line^27–29^. Within the context of this debate, our experiments indicate that the roughness of borosilicate glass vials (measured RMS value of $\sim 1$ $\upmu$m by confocal profilometry) induces triple line nucleation, leading to dendrite growth at the meniscus-glass contact line. After nucleation, dendrites grow with a velocity of $\sim$ $15$ cm/s, matching literature values for dendritic ice crystallization on substrates or in confined spaces^19,20^. Dendritic crystallization in bulk water has been reported to be significantly slower^30^. The thermal conductivity of glass exceeds that of pure water, resulting in a temperature gradient that extends from the colder glass interface to the warmer liquid center. Consequently, the water near the glass interface experiences the highest degree of undercooling, promoting dendritic growth along the glass interface.

When the freezer is opened at the stage where the dendrites have formed, one can still pour out the liquid in the center of the vial and observe that the ice dendrites indeed form a thin layer near the walls. This means that sample is opaque yet remains blue in transmission because there is only a thin layer of dendritic ice formed on the glass interface. If the opaque sample is left at $-30$ $^\circ$C, nucleation and growth of transparent ice follows in a second step and the sample becomes colorless. The latter initiates again at the contact line of the concave meniscus (shown with a red arrow at 1500 s in Fig. 1). The water/ice phase transition progresses at the air/liquid meniscus and from the glass wall of the container towards the central part of the vial; the freezing front of ice can be visualized as the boundary between colorless ice and the blue liquid phase.

As long as the meniscus is not completely frozen, due to the volumetric expansion of ice, the remaining liquid water can escape towards the air (see the movie 1 in the Supplementary Material) from the opening in the ice/air interface. When the meniscus is completely frozen, the remaining liquid water gets entrapped in the heart of the growing ice. When the trapped liquid pocket freezes, this leads (in this experiment after 2640 s) firstly to the fracture of the ice (having weaker mechanical properties than the glass) followed by the fracture of the glass container.

In total, we observe such a two-step crystallization process of ice in 65 out of 90 untreated glass samples. For the rest of the samples, the growth of transparent ice occurs directly during the phase transition without passing through the first step of dendritic growth. High-speed-camera observations combined with measurements of the glass temperature (Fig. 2a) confirm the correlation between dendritic crystallization and the supercooling. Dendritic ice forms when freezing occurs at sufficient supercooling, accompanied by a temperature jump. The blue curve in Fig. 2a indicates a $\Delta T \approx$ 6 $^\circ$C at the moment dendrites are observed, as depicted by the asterisk. Conversely, the slope of the green curve decreases without a distinct $\Delta T$ for a glass temperature of about $-6$ $^\circ$C. Since no dendrites were observed at that moment we infer that the water is near $T_m$, resulting in bulk crystallization.

Surprisingly, the emergence of dendritic crystallization preceding bulk crystallization was found to affect the probability of fracture. Statistical analysis indicates that out of the 54 glass containers showing dendritic crystallization, $53.7 \%$ fractured. Conversely, among the 36 containers that crystallized directly, $83.3 \%$ fractured. Inspection of the samples reveals a significant increase in the number of entrapped air bubbles for the samples that underwent dendritic crystallization followed by the bulk ice formation (Fig. 2b). A likely explanation seems to be that the entrapped air bubbles increase the effective compressibility of the ice/air system and in this way attenuate the stress resulting from the volume expansion when the liquid inclusion turns to ice^31^. This in turn suggests that the presence of air bubbles can play a crucial role in reducing the fracture of confined growing ice.

### Entrapment of the liquid inclusion

To investigate the dynamics of liquid inclusion formation, we tracked the boundary of the blue liquid phase in time. By assuming the inclusion is radially symmetric, we can approximate its volume *V* by a body of revolution rotating around the vertical axis. The body is divided into multiple slabs one pixel high before each slab is rotated individually around the central axis (Fig. 3a). Figure 3b shows the temporal evolution (extracted every 2 s) of the equivalent radius *R* of the approximated liquid inclusion volume given by $V =4/3 \pi R^3$. The vertical dashed lines in Fig. 3b show the moment when the meniscus was fully frozen ($t_n$). Among the 90 vials subjected to examination, 59 were broken, while the remaining 31 remained intact.

To examine the propagation rate of the ice front when the liquid pocket has formed, we plot the radius of the inclusion against the time ($t_0 - t$) in the inset of Fig 3b. Herein, $t_0$ is the time at which the inclusion is completely crystallized. This allows us to determine the freezing rate from different experiments giving a different $t_0$ in situations where no liquid can escape, and consequently, only crystallization contributes to the reduction in liquid volume. In the cases where no fracture occurred, we find that the radius of the inclusion scales approximately with the square root of time ($r\sim \sqrt{t_0-t}$), as expected for a process in which the diffusion of latent heat of crystallization is the limiting factor for the freezing speed. To be more precise, a power law fit $r=a(t_0-t)^b$ applied on the blue and green data yields a power of $b = 0.50 \pm 0.013$ and $b = 0.52 \pm 0.015$, respectively. Here, $t_0$ is not a free fitting parameter but the observed time at which all the liquid has crystallized. Remarkably, when fracture does occur, the radius does not obey the predicted shrinking rate in time. We find an exponent of $b = 0.35 \pm 0.023$ for the red data, indicating that ice crystallization before fracture is not only limited only by diffusion, but that the stress buildup also affects the dynamics. Another notable observation in the red data is the discontinuous decrease in liquid volume at the moment of fracture. Upon fracture, the pressure drops from several hundred MPa (calculation provided below) to ambient pressure. This pressure drop leads to an increase in melting temperature: $\Delta T_{\text {m}} \sim 1^\circ$C each 12 MPa^32^. Consequently, crystallization is significantly enhanced at the moment of fracture.

To see what the pressure build-up due to the inclusion is, we need to do the thermodynamics and mechanics of the problem. In 1952, Gulida and Lifshitz^25^ examined the thermodynamics of local melting. This is mentioned in a footnote in some editions of the Theoretical Physics series of Landau & Lifshitz, but the original article is in Russian. We add our translation of the paper as an Appendix. The problem posed is that of the local melting of a solid, e.g. by local heating. Since for most substances, the liquid is less dense than the solid phase, local melting (e.g. by a localized heating source within the solid) will lead to a buildup of pressure due to volume expansion, which shifts the melting temperature since the pressure changes the chemical potential of liquid and solid differently. We are here in exactly the same situation since for water the ice is less dense than the liquid, and one can thus use the same calculation to calculate the pressure that the liquid pocket exerts upon the ice when it crystallizes. Transposing Gulida and Lifshitz’ calculation to our case, the pressure exerted at the liquid-solid interface upon freezing of the trapped water, assuming that the liquid inclusion is homogeneous, is:

1 $$\begin{aligned} P=\frac{(4\mu +3k_1)k_2 P_0-4\frac{\delta \rho }{\rho _w}\mu k_1 k_2}{k_1(4\mu +3k_2)} \end{aligned}$$

**Figure 4:**
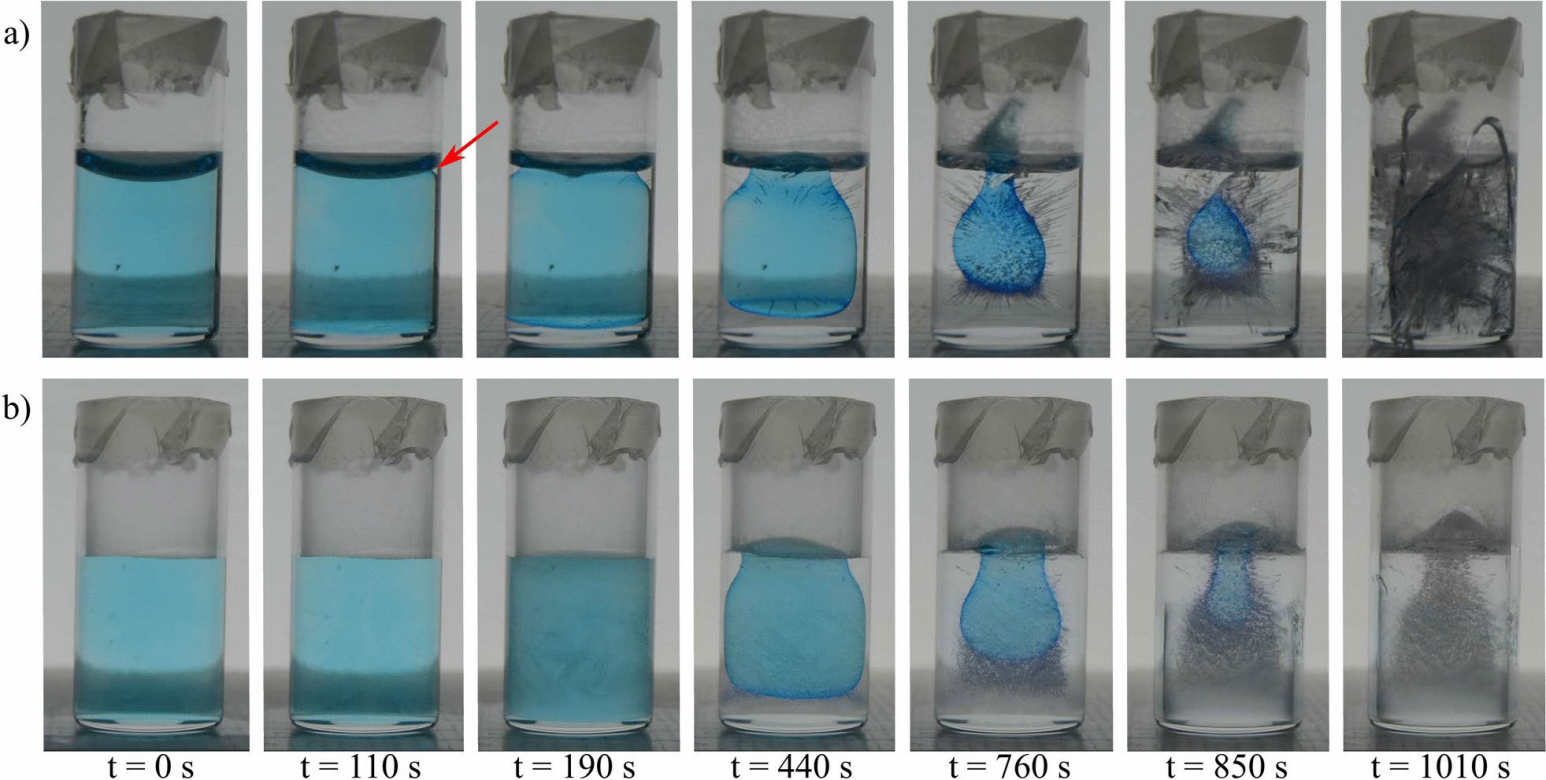
Snapshots of key moments in the freezing process of 2 mL confined water at an ambient temperature of $-30$ $^\circ$C in two different recipients: **(a)** hydrophilic, where the liquid gets enclosed and eventually causes fracture in both ice and glass. This sample corresponds to red dots in Fig. 3b, c. Red arrow indicates the location where bulk crystallization initiates, at the contact line of the concave meniscus. **(b)** Hydrophobic, demonstrating the absence of a liquid inclusion, therefore no pressure is generated and leaving both the ice and glass intact. This sample corresponds to the red triangles in Fig. 3c.

where $\mu \sim 2.5-3.5$ GPa is the shear modulus of ice^33,34^, $k_1 \sim 2.1$ GPa and $k_2 \sim 10$ GPa represent the bulk moduli of water and ice, respectively, $P_0 \sim 0.101$ MPa is the ambient pressure, $\delta \rho \sim 90$ kg/m$^\text {3}$ the difference in density between ice and liquid water, and $\rho _w \sim 997$ kg/m$^\text {3}$ the water density. By assuming the water inclusion is an isotropic sphere, Eq. (1) can be applied to calculate the exerted pressure; we find $P \sim 260 \pm 30$ MPa for our experimental parameters. Note the independence of pressure on the inclusion boundary from the enclosed liquid volume. A recent study has found similar results for supercooled droplets frozen from the outside. In cases where the formed crystalline shells are sufficiently thick, the pressure build-up is also invariant with respect to the liquid radius^35^. Alternatively, Eq. (1) can be simplified. The first term of the numerator is orders of magnitude smaller, and could be neglected:

2 $$\begin{aligned} -\frac{4\delta \rho \cdot \mu k_2}{\rho _w(4\mu +3k_2)}=-\frac{\delta \rho }{\rho _w}\frac{2E_2}{9(1-\nu _2)}, \end{aligned}$$

with Young’s modulus $E_2 \sim 9.1$ GPa and the Poisson ratio $\nu _2 \sim 0.33$ of ice, respectively^36^. This expression, in line with Eshelby inclusion theory^37^, is perhaps a more intuitive representation of the physics and yields $P\sim 270$ MPa.

The next step is to estimate the pressure that our borosilicate vials can support; this can be computed using Griffith’s criterion for linear elastic fracture mechanics^38^:

3 $$\begin{aligned} \sigma = \sqrt{\frac{2 E \gamma }{\pi a}}. \end{aligned}$$

With Young’s modulus $E = 62 \times 10^9$ Pa, surface energy $\gamma = 55 \times 10^{-3}$ J/m$^2$ and an internal flaw diameter $a = 0.3 - 1$ $\upmu$m^39^, we find a maximum pressure of $\sim 65 \pm 20$ MPa, which is in good agreement with experimental literature^40^. Hence, the built up pressure generated by a volumetric increase during freezing exceeds the required pressure to break a glass confinement. The tensile stress of ice, influenced by factors such as temperature and grain size distribution, is in the order of a few MPa^41,42^, approximately one order of magnitude lower than that of borosilicate glass. Therefore, we generally observe the first cracks within the ice in the tangential direction (as shown in Fig. 1a) before the fracture of the glass vial.

### Wettability of the container and neck closure

To examine the effect of wettability on the freezing process, we studied the freezing dynamics in three types of glass vials with varying degrees of wettability (supplementary Fig. S1) as described in the Methods section. We refer to these as hydrophilic, untreated vials in which water meniscus is concave and hydrophobic confinements with a flat meniscus. The freezing experiments in these vials reveal the significant role of the location of ice nucleation in the final mechanical damage observed; in both hydrophilic and untreated containers, ice nucleation occurs at the contact line of the concave meniscus. On the other hand, in the hydrophobic recipients, as the ’wedge effect’ is almost inexistent for a flat meniscus, nucleation is observed to occur rather at the bottom wall of the glass container. Consequently, while ice growth progresses from the glass wall toward the central part of the container, water can escape for a longer time in the unconfined direction from the unfrozen meniscus (Fig. 4, or movie 2 and 3 in supplementary). It is interesting to note that the speed of the freezing of the meniscus is found to be constant at approximately 0.017 mm/s, regardless of the wettability, i.e. the shape of the meniscus (Fig. 3c). As a result, there is no entrapped liquid inclusion in hydrophobic vials, which explains the absence of fracturing in the hydrophobic recipients; a total of 22 hydrophobic samples were examined, all of which initiated ice nucleation at the bottom of the glass. Consequently, none of the hydrophobic samples exhibited entrapped liquid inclusions, and no fractures were observed in either the ice or the glass.

**Figure 5:**
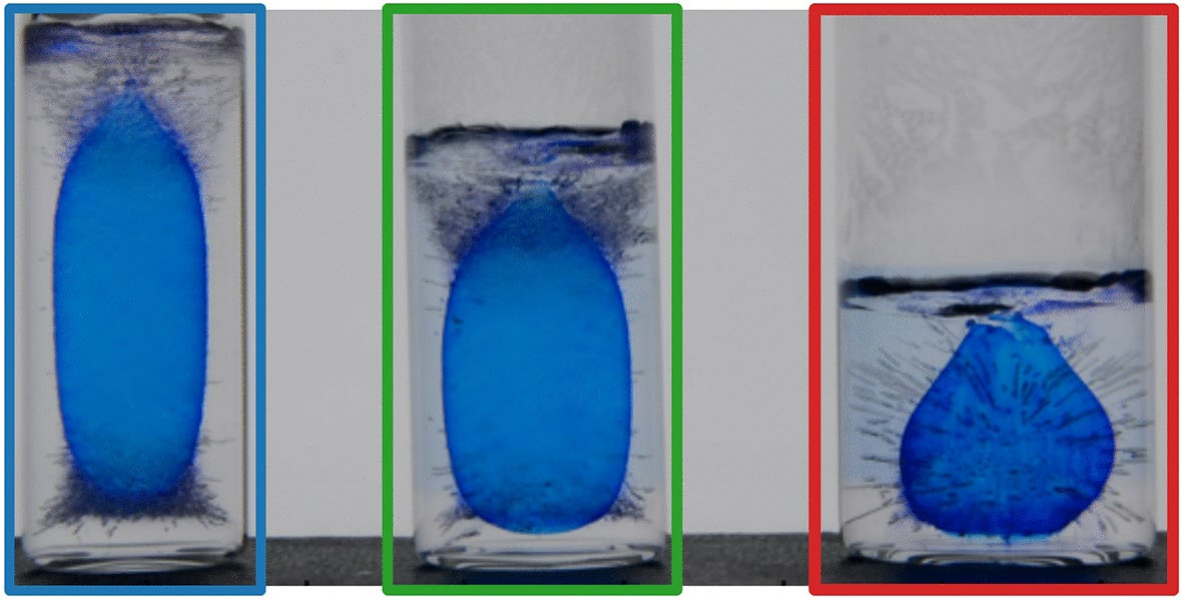
Liquid inclusion formation in ice in three glass container used to investigate the effect of the radius-to-volume ratio on the fracture probability. In blue 6.35 mm, in green 7.35 mm, in red 9.35 mm. Images show the moment at which the meniscus freezes completely and the remaining liquid becomes fully enclosed. Colors correspond to the colors in Fig. 6.

### Radius of the vials

**Figure 6:**
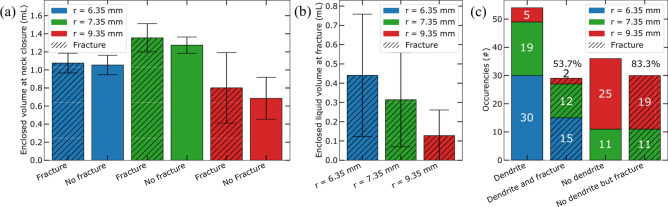
**(a)** Average entrapped liquid volume at the moment of meniscus closure (cf. Fig. 5) for glass containers of three different radii. Hatched bars indicate containers that fractured during the experiment whereas non-hatched bars indicate containers that did not. Error bars represent the standard deviation. **(b)** Average enclosed liquid volume at the moment of fracture. The large error bars indicate that there is no clear threshold in the amount of expansion for fracture to occur. **(c)** Statistics on the observation of dendritic crystallization. 54 samples exhibited dendrites of which $53.7 \%$ fractured. 36 samples did not exhibit dendritic crystallization, leading to a higher fracture probability of $83.3 \%$. Remarkably, all small containers (in blue) underwent dendritic crystallization before bulk crystallization.

The effect of the surface-to-volume ratio of the confinement was also examined by performing experiments on three cylindrical recipients with identical glass thickness (1 mm) but varying the inner radius (6.35, 7.35, and 9.35 mm). The volume of the water was kept constant at 4 mL. Fig. 6a shows the enclosed liquid volume at the moment of meniscus closure for the different sized recipients. The variation in shape results in an average encapsulated liquid volume at meniscus closure of $1.06 \pm 0.11$, $1.34 \pm 0.15$, and $0.77 \pm 0.35$ mL for the containers with 6.35, 7.35, and 9.35 mm radius, respectively. To examine an influence of the size of the inclusion on fracture probability we categorized the data in two groups: vials that fractured and those that did not. We do not find a statistically significant correlation between the enclosed liquid volume at meniscus closure and the probability of fracture. In addition, at the moment of fracture, the amount of liquid remaining in the inclusion (Fig. 6b) was quantified. The latter varies widely resulting in large error bars. This again suggests that there is no specific threshold volume expansion value after which fracture occurs, in agreement with the mechanical arguments presented above.

The glass recipients with the smallest inner radii exhibit a higher frequency of dendritic crystallization prior to bulk ice crystallization (Fig. 6c). This is likely due to the higher surface-to-volume ratio of the smaller containers, which leads to a greater degree of supercooling and, consequently, a higher probability of dendritic growth. As a result, we observe a clear negative correlation between dendritic crystallization and fracture probability: $53.7 \%$ of the 54 samples fractured. Conversely, 36 samples did not exhibit dendritic crystallization, and $83.3 \%$ of these samples fractured. In addition, the shape of the entrapped liquid inclusion beneath the complete frozen meniscus varies in different vials (Fig. 5). The wider the cylinder, the more spherical the shape of the liquid inclusion.

## Discussion

We conducted an experimental investigation to elucidate the freezing kinetics and the mechanism of freezing-induced damage in partially saturated media. We demonstrate that the condition to induce damage is the formation of a liquid inclusion during freezing in bulk ice; when this inclusion freezes, the $\sim$ $9\%$ volume expansion of water during the phase transition to ice causes the damage. By the addition of methylene blue to water, we have been able to visualize ice front propagation and the kinetics of liquid inclusion formation. The subsequent pressure build-up upon its conversion to ice was calculated to reach $\sim$260 MPa, which is indeed large enough to break the glass container walls. Consistent with the theoretical prediction that pressure on the inclusion boundary is independent of the volume of the liquid inclusion, we observe no significant difference in inclusion size and fracture probability (Fig. 6a). Furthermore, the wide variation in enclosed liquid volume present at the moment of fracturing (Fig. 6b) highlights the lack of correlation between entrapped liquid volume and fracture risk.

We demonstrate that hydrophobic coatings can prevent the formation of liquid inclusions by increasing the contact angle of the meniscus, which delays crystallization at the water/air interface. This finding aligns with recent work by Bi et al., who showed that geometric constraints such as those imposed by wedges, limit the movement of water molecules and enhance ice crystallization by aligning the molecules with the ice structure^43^. This effect is similar to lowering the entropy-related component of the free energy barrier. The authors also propose that the traditional concept of misfit strain between a nucleation site and a crystalline lattice should be expanded to include unconventional surface topographies. Our findings support this idea: the water/air meniscus, with its contact angle against the glass wall, acts as a geometric constraint similar to wedges, which promotes ice nucleation in conventional hydrophilic glass containers.

We also found that the size of the confinement can affect the freezing dynamics and crystallization process. Smaller glass containers induces larger supercooling; these exhibit a more frequent occurrence of dendritic crystallization prior to bulk ice crystallization. The presence of dendritic ice growth at the glass-liquid surface was found to negatively correlate with the fracture probability. This is likely due to large amount of entrapped air bubbles that appeared in the bulk ice crystal for samples that first underwent dendritic crystallization. Fast-growing dendrites can trap dissolved gas that is expelled from the crystalline structure, leading to increased bubble formation during bulk freezing^44^. The presence of these bubbles enhances heterogeneous nucleation, and local changes in pressure and surface energy during bulk freezing may facilitate further cavitation^45^. As a consequence, the compressive strength of ice decreases, leading to the absorption of the stress generated by the expansion of the liquid inclusion while converting to ice. The recipe to prevent catastrophes in your freezer is therefore to have small and hydrophobic bottles.

## Methods

The experimental setup consisted of a climate chamber (Weiss KWP 240) with a window that allowed for observation. For the freezing experiments the temperature was mostly maintained at a fixed value of $-30^\circ$C. Glass vials of different sizes were subjected to a rigorous cleaning process with ethanol and deionized water (Millipore, $\rho \approx 18.2$ M$\Omega \cdot$cm) to ensure the removal of any contaminants. Following the cleaning process, the vials were filled with 4 mL of deionized water, which had been degassed in a vacuum chamber for 2 hours. To visualize the propagation of the ice front, we add 8 mg/L methylene blue, a blue dye that is very soluble in water. As with any impurity, it gets expelled by the crystal lattice during the liquid/solid phase transition. And even if it gets entrapped in the ice due to its rapid growth, it forms dimers and tetramers that are not colored; therefore it exclusively probes the liquid phase in blue^46–48^, which allows for the unambiguous detection of the liquid /ice interface during freezing experiments. We checked that the addition of methylene blue did not in any way influence the crystallization location or dynamics, by repeating the experiment without the dye (Fig. S2). Prior to being placed inside the climate chamber, the vials, although open, were covered with Parafilm to minimize possible impacts of dust and changes in humidity. To investigate the effect of the geometry, we used glass vials with three inner radii of 6.35, 7.35, and 9.35 mm. The glass thickness of all vials was 1 mm as measured with a confocal profilometer (Keyence VK-X1100). Glass containers with different wetting properties were used to study the effect of surface hydrophobicity or hydrophilicity on the freezing process. To have a hydrophobic surface, glass vials were washed with water and isopropanol, dried, cleaned by plasma treatment (30 s), and submerged into a solution of toluene and trichlorooctylsilane (from Tokyo Chemical Industry, 1$\%$ vol) for 15 minutes. They were subsequently generously rinsed with isopropanol and water. For a hydrophilic surface, after the washing step, glass vials were treated with an air plasma for 1 minute. The effect of the treatments results in different shapes of the liquid meniscus (flat or concave) since it affects the contact angle of the air/water meniscus on the glass wall, as shown in supplementary Fig. S1. Contact angles of $90\pm 3.6^{\circ }$, $79\pm 2.1^{\circ }$, and $68\pm 2.6^{\circ }$ were obtained for hydrophobic, untreated, and hydrophilic containers, respectively. A Nikon D5200 camera and a Phantom TMX 7510 high-speed camera were used for taking temporal images of the water/ice phase transition during freezing. Thirty measurements were performed for each vial size or wettability property to allow for a statistical analysis in terms of the probability of fracturing. In total more than 120 experiments were been performed for this study.

## Supplementary Information

Supplementary Information 1.

Supplementary Video 1.

Supplementary Video 2.

Supplementary Video 3.

## Data Availability

All data are available in the main text or the supplementary materials, for further needs contact corresponding author.
