# Supplementary material for: Damage due to ice crystallization
Source: Sci Rep. 2025 Jan 16;15:2179. doi: 10.1038/s41598-025-86117-5 (PMC11739483; doi:10.1038/s41598-025-86117-5)
Supplement: Supplementary file 1 — Supplementary Information 1. [file 41598_2025_86117_MOESM1_ESM.pdf]

Supplementary Information
Damage due to Ice Crystallization

Menno Demmenie,[1,2*] Paul Kolpakov,[1] Boaz van Casteren,[1] Dirk Bakker,[1] Daniel Bonn,[1] Noushine Shahidzadeh ,[1]

[1] Van der Waals–Zeeman institute, Institute of Physics, University of Amsterdam, Science Park 904, 1098 XH Amsterdam, The Netherlands.

[2] Van 't Hoff Institute for Molecular Sciences, University of Amsterdam, Science Park 904, 1098XH Amsterdam, The Netherlands

E-mail: M.Demmenie@uva.nl.

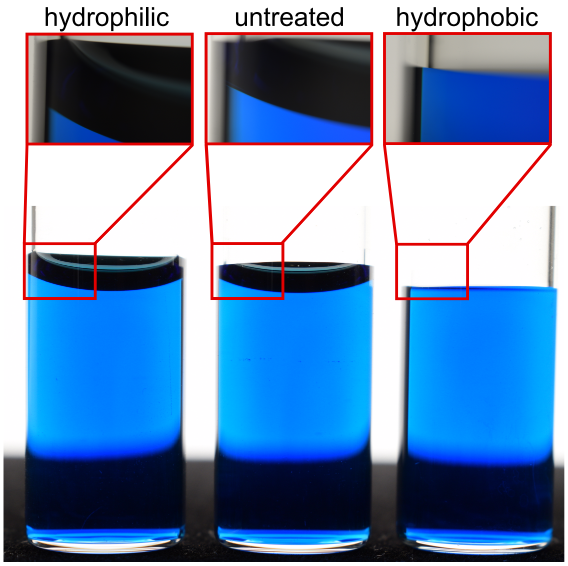

Figure S1: The effect of glass wettability treatments on the meniscus of water inside glass vials. The contact angle of the menisci of hydrophilically treated (θ_{hpl}), untreated (θ_{ut}) and hydrophobically treated (θ_{hpb}) recipients yielded values of $68 \pm 2.6°$, $79 \pm 2.1°$ and $90 \pm 3.6°$ at $t = 0$, respectively.

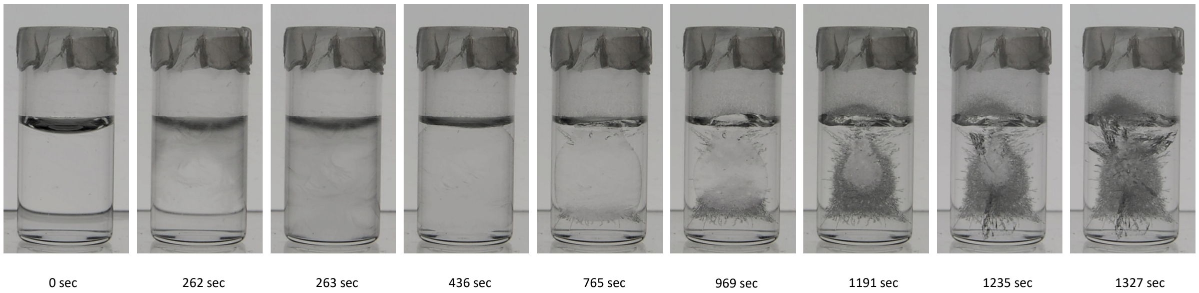

Figure S2: Freezing Dynamics of 2 mL MilliQ Water at -30°C in an Untreated Recipient, with the omission of methylene blue dye.

1 Appendix

What follows below is a translation of the iconic paper ”On theory of local melting” written by I. Lifshitz and L. Gulida.

When considering the process of melting solids, it is usually assumed that pressures and temperatures remain constant along the solid phase. In the case of a temperature gradient or an inhomogeneous stress state in a solid, one can expect the appearance of melting centers inside the solid phase with their subsequent development into local regions of the liquid phase. Let, for example, a heat source be located inside a solid body, and such a temperature is maintained at its boundary that excludes the possibility of melting from the surface. However, in some areas inside the solid phase, the temperature can be so high that it becomes possible for the appearance of local melting centers that arise in a fluctuation way.

Assuming the size of the nuclei to be small compared to the distances at which the temperature T or stresses σ_{ik} change noticeably, it will suffice to consider the appearance of a single liquid nucleus in an unlimited solid phase homogeneous with respect to T and σ_{ik}. The choice of the potential ϕ, which describes the state of the system, is, to a certain extent, arbitrary, since the nature of the conditions specified on the surface, which is infinitely distant relative to the nucleus, is insignificant.

One can, for example, set a uniform external pressure P_0 on this surface and describe the system by the thermodynamic potential Φ or fix the volume of the body, characterizing the state of free energy F. Then, $\Delta\Phi$ in the first case and ΔF in the second case will coincide. In what follows, for definiteness, we will assume that the external pressure is fixed.

We represent $\Delta\Phi$ as the sum of three terms:

$$\Delta\Phi = \Delta\Phi_{heat} + \Delta\Phi_{vol} + \alpha\sigma, \tag{1}$$

$\Delta\Phi_{heat}$ is due to the change in phase stage at P_0; $\Delta\phi_{vol}$ is due to the change in strain energy and volume of the system; α is the surface tension coefficient at the interface between the solid and liquid phases; σ is the nucleation surface area.

Denoting by T the actual temperature of the solid phase, and by T_0 the melting temperature at $P_0 = 0$ and expanding $\Delta\Phi_{heat}$ in a series in steps $T - T_0$, we obtain:

$$\Delta\Phi_{heat} = -\frac{q}{T}(T - T_0)v, \tag{2}$$

Where q is the heat of fusion at $T = T_0$, calculated per unit volume, and v is the volume of the liquid nucleus.

We write $\Delta\Phi_{vol}$ as follows:

$$\Delta\Phi_{vol} = \frac{P^2}{2k_2}v + \int_{V-v} w dv + P_0\Delta V - w_0 V, \tag{3}$$

Where k_1 and k_2 are volume compression moduli of the solid and liquid phases; P the pressure occurring at the surface of the liquid nucleus; $w_0 = P_0^2/2k_1$ and w are energy densities of deformation of solid phase in initial and final states; V is initial volume of system; and ΔV is the change in volume of the whole system caused by the formation of the nucleus.

w and P at given values of P_0 and volume v depend on the shape of nucleus surface. This shape must be determined from the condition of a minimum potential Φ. Thus, in order to actually deduce $\Delta\Phi$ with equation 1, a complex variational problem has to be solved.

The pressure P on the surface of the nucleus is determined from the equation

$$P = -k_2\left(\frac{v_l(P) - v_l(0)}{v_l(0)}\right) = -k_2\left(\frac{\delta v}{v_s(0)} + \frac{\delta\rho}{\rho_s}\right) \tag{4}$$

Here v_l and v_s are the volumes of particles forming the nucleus in the liquid and solid phase at the corresponding pressures; ρ_l and ρ_s are the densities of the liquid and solid phases; $\delta\rho = \rho_l - \rho_s$. It is assumed that $\delta\rho \ll \rho_s$. $\delta_v = \delta_v(P)$ can be interpreted as a change in the volume of the cavity occupied by the liquid nucleus when a uniform pressure P is applied to its boundary. δ_v is determined by solving basic equations of elasticity theory under the following boundary conditions:

$$\sigma_n = -P \text{ at the nucleus surface,} \tag{5a}$$

$$\sigma_n = -P_0 \text{ at the infinitely distant surface,} \tag{5b}$$

where σ_n is the normal stress.

In an isotropic solid phase under uniform external pressure, the liquid nucleus will be confined to a sphere. The radial displacement u_R satisfying equation 5 is:

$$u_R = \left(a + \frac{b}{R^3}\right) R, a = \frac{-P_0}{3k_1}, b = \frac{(P - P_0)R_0^3}{4\mu}, \tag{6}$$

with R_0 the nucleus radius and μ the shear modulus.

Using equation 3 and 4 and we find that

$$P = -k_2 \left(\frac{3u_{R_0}}{R_0} + \frac{\delta\rho}{\rho_s}\right) = \frac{(4\mu + 3k_1)k_2 P_0 - 4\frac{\delta\rho}{\rho_w}\mu k_1 k_2}{k_1(4\mu + 3k_2)}, \tag{7}$$

$$\Delta\Phi = \left(\Psi - \frac{q\Delta T}{T_0}\right) v + 4\pi R_0^2 \alpha \tag{8}$$

Where

$$\Psi = \Psi(P_0) = \frac{(k_2 - k_1)(4\mu + 3k_1)P_0^2}{2k_1^2(4\mu + 3k_2)} - \frac{k_2(4\mu + 3k_1)\delta\rho P_0}{k_1(4\mu + 3k_2)\rho_s} + \frac{2\mu k_2}{4\mu + 3k_2}\left(\frac{\delta\rho}{\rho_s}\right)^2. \tag{9}$$

Using expression 8, it is possible to determine at which external pressure local melting is possible if the temperature is fixed.

For a given $\Delta T = (T - T_0)$ the possible values of P_0 are determined by the condition:

$$\Psi(P_0) - \frac{q}{T_0}\Delta T \ll 0. \tag{10}$$

The curves corresponding to equation 10 are parabolas directed upwards for $k_2 < k_1$ and downwards for $k_2 > k_1$.

If $k_2 < k_1$, then local melting is possible at pressure P_0, or greater than P_2, or less than P_1, where P_1 and P_2 are roots of equation 10.

if $k_2 > k_1$, then local melting is possible at $P_1 < P_0 < P_2$, when $\delta\rho < 0$ and $\Delta T \to 0$ roots of $P_{1,2}$ can be negative. In this case, local melting is possible only under conditions of all-round stretching. If ΔT is negative and large, then the roots of equation 10 will be complex. At such temperatures, local melting is generally impossible.

The temperature T, corresponding to the beginning of local melting, will also be determined from equation 10, in which P_0 should be considered fixed:

$$\Delta T_{loc} = \frac{\Psi}{q} T_0 \tag{11}$$

Expanding in the ratio

$$\Phi_{liq}(P_0, T) - \Phi_{sol}(P_0, T) = 0 \tag{12}$$

in potentials of each of the phases in a series in powers of $T - T_0$ and P_0, we obtain the dependence of the usual melting temperature on pressure:

$$\Delta T_{usual} = \frac{T_0}{q}\left[\frac{k_2 - k_1}{2k_1k_2}P_0^2 - \left(\frac{\delta\rho}{\rho_s}\right)P_0\right]. \tag{13}$$

Curves 11 and 13 are parabolas with tangent points at $P_0 = \frac{k_1 k_2 \delta\rho}{\rho(k_2 - k_1)}$, and $\Delta T_{loc} - \Delta T_{usual} > 0$. Thus, local melting occurs under the conditions of overheating of the solid phase (at fixed P_0 and T). The dimensions of the liquid nucleus are determined from the equation $\frac{d\Delta\Phi}{dR_0} = 0$ where,

$$R_0 = \frac{-2\alpha}{\Psi - \frac{\Delta T}{T_0} q}. \tag{14}$$

Using equation 8 and 14 we find that the probability of the formation of a nucleus:

$$W \sim e^{\Delta\Phi/kt} = exp\left[\frac{-2\pi(2\alpha)^3}{3kT(\Psi - \frac{\Delta T}{T_0} q)^2}\right]. \tag{15}$$

Expressions 8 and 14 for $\Delta\Phi$ were obtained under the assumption that the deformations are purely elastic. If $k_2 - k_1$ or $\delta\rho$ become large enough, then plastic deformations will appear in the solid phase.

Using equation 3 and 4 and the well-known solution of the problem of equilibrium of an elastic-plastic spherical shell, it is possible to obtain the equation for determining P:

$$\left(\frac{k_2}{k_1} - 1\right) P \mp \frac{k_2}{\alpha} e^{\frac{\sqrt{3}}{2}\frac{P-P_0}{k_2} - 1} - k_2 \frac{\delta\rho}{\rho_s} = 0, \tag{16}$$

and $\Delta\Phi_{usual}$ will be equal to:

$$\begin{aligned} &\Delta\Phi_{usual} = \frac{P^2}{2k_2} - \frac{P_0^2}{2k_1} + \frac{3}{8\mu}(P_1^2 - P_0^2) + \\ &\frac{P_1^2 - P_0^2}{2k_1} + \frac{1}{\alpha_1}\left(\pm P - \frac{2k_3}{\sqrt{3}}\right) \cdot \\ &exp\left(\pm\frac{\sqrt{3}}{2}\frac{P - P_0}{k_3} - 1\right) \mp \frac{P_0}{\alpha_1} - \frac{\Delta T}{T_0} q, \end{aligned} \tag{17}$$

where k_3 is the yield point; $\alpha_1 = \frac{2\sqrt{3}\mu k_1}{k_3(4\mu + 3k_1)}$, $P_1 = P_0 \pm \frac{2k_3}{\sqrt{(3)}}$ is the value of P at which a plastic interlayer appears. The upper sign in 16 and 17 should be taken at $P - P_0 > 0$; the lower one at $P - P_0 < 0$. It follows from the consideration of relation 17 that for $k_2 = k_1$ and $\delta\rho \neq 0$ i.e. $\Delta T_{loc} - \Delta T_{usual} > 0$ and taking into account the plastic character of deformation, local melting begins under overheating conditions. For metals, relation 12 gives overestimated

temperatures for the onset of local melting. It is possible to indicate other particular cases when $\Delta T_{loc} - \Delta T_{usual} > 0$. Without exact knowledge of the root of equation 16, however, it is impossible to assert that this inequality will be satisfied or any values of k_2, k_1 and $\delta\rho$.

In conclusion, we note that, from the point of view of the hypothesis of local melting, it seems possible to interpret the experiments of S. E. Khaikin and N. P. Binet, who studied the process of pressing tin rods under conditions of an artificially created temperature gradient. In polycrystalline samples, melting was observed, starting inside the solid phase, while in single crystals of tin k, overheating of $1.5 - 2$°C was observed.
